# A novel interlamellar oral mucosa graft surgery technique using fibrin glue for the treatment of trichiasis

**DOI:** 10.5935/0004-2749.20230010

**Published:** 2023

**Authors:** William W. Binotti, Ana Carla B. de Aguiar, Aline A. Barbosa, Sergio V. Burnier

**Affiliations:** 1 Ophthalmology Department, Complexo Hospitalar Ouro Verde “Prefeito Edivaldo Orsi”, Campinas, SP, Brazil

**Keywords:** Trichiasis, Interlamellar surgery, Van Millingen surgery, Fibrin glue, Biological glue, Oral mucosal graft, Triquíase, Cirurgia interlamelar, Cirurgia de Van Millingen, Cola de fibrina, Cola biológica, Enxerto de mucosa oral

## Abstract

**Purpose:**

The purpose of this study was to evaluate the long-term outcomes of patients with trichiasis treated with a modified interlamellar oral mucosa graft surgery technique using fibrin glue.

**Methods:**

A prospective study was conducted at the Oculoplastic Department of Ouro Verde Hospital Complex. Patients with recurrent trichiasis without entropion who did not respond to conventional therapy, underwent intermarginal lamellar splitting of the eyelid and oral mucous graft insertion with fibrin glue replacing sutures. They were then evaluated at 1-day, 1-week, 1-month, 6-month, and 4-year follow-ups. Graft adherence, symptom resolution, esthetic satisfaction, overall patient satisfaction, and trichiasis recurrence were assessed at 6-month and 4-year follow-ups.

**Results:**

Fifteen patients (a total of 19 eyes) were included, of whom 10 (66.7%) were female and 5 (33.3%) were male. The mean age was 75.4 ± 10.5 years (range, 54-98 years). Acquired trichiasis was the main cause. Of the patients with acquired trichiasis, 12 (86.7%) had chronic blepharitis, 2 (13.3%) had an undetermined cause, and one (6.7%) had trachomatous trichiasis. Most cases involved only one eyelid segment (89.4%) and =5 lashes (84.2%; minor trichiasis). No adverse reactions from the fibrin glue were reported and no sutures were required after graft placement. At 6 months, no graft failures occurred, 17 eyes of 13 patients (89.4%) showed good graft adherence, 2 eyes of 2 patients (10.5%) showed partial graft adherence, and 2 eyes of 1 patient (10.5%) had trichiasis recurrence. At 4-year follow-up, no graft failure occurred, 3 patients (3 eyes) were lost to follow-up, and 2 eyes of 2 patients (14.2%) had trichiasis recurrence. The 4-year cumulative success rate was 78.9%.

**Conclusions:**

The modified interlamellar surgery with fibrin glue showed a good long-term success rate. This technique reduces surgical time, facilitates smaller graft insertion, and therefore, should be considered for recalcitrant minor trichiasis without entropion.>

## Abstract (pt)

**Objetivo:**

O objetivo deste estudo foi avaliar os resultados a longo prazo da técnica cirúrgica modificada de enxerto interlamelar de mucosa oral usando cola de fibrina para o tratamento de triquíase.

**Métodos:**

Um estudo prospectivo foi realizado no Departamento de Óculo-plástica do Complexo Hospital Ouro Verde. Pacientes com triquíase recorrente sem entrópio, que não responderam à terapia convencional, foram submetidos à cirurgia com separação intermarginal das lamelas das pálpebras e inserção de enxerto de mucosa oral usando cola de fibrina, substituindo a sutura. Pacientes foram avaliados 1 dia, 7 dias, 1 mês, 6 meses e 4 anos após a cirurgia. A aderência do enxerto, resolução dos sintomas, satisfação estética, satisfação geral do paciente e recorrência de triquíase foram avaliados aos 6 meses e aos 4 anos.

**Resultados:**

Quinze pacientes (total de 19 olhos) foram incluídos, dos quais 10 (66.7%) eram do sexo feminino e 5 (33.3%) do sexo masculino. A média de idade foi 75.4 ± 10.5 anos (intervalo 54-98 anos). Triquíase adquirida foi a principal causa, da qual 12 pacientes apresentaram blefarite crônica (86.7%), 2 pacientes com causa indeterminada (13.3%) e 1 paciente com triquíase tracomatosa (6.7%). A maioria dos casos envolveu apenas um segmento da pálpebra (89.4%) e com =5 cílios (84.2%; triquíase menor). Nenhuma reação adversa foi reportada com o uso da cola de fibrina e nenhum caso necessitou de sutura após inserção do enxerto. Aos 6 meses, não houve nenhuma falha de enxerto, 17 olhos de 13 pacientes (89.4%) apresentaram boa aderência de enxerto, 2 olhos de 2 pacientes (10.5%) mostraram aderência parcial do enxerto e 2 olhos de 1 paciente (10.5%) apresentaram recorrência da triquíase. Aos 4 anos, não houve nenhuma falha de enxerto, 3 olhos de 3 pacientes tiveram perda de seguimento e 2 olhos de 2 pacientes (14.2%) apresentaram recorrência da triquíase. A taxa de sucesso acumulativa após 4 anos foi de 78.9%.

**Conclusão:**

A cirurgia modificada de enxerto interlamelar de mucosa oral usando cola de fibrina mostrou uma boa taxa de sucesso a longo prazo. Esta técnica reduz o tempo cirúrgico, facilita a inserção de enxertos menores e, portanto, deve ser considerada em triquíase menor sem entrópio resistente ao tratamento convencional.

## INTRODUCTION

Trachoma is the leading infectious cause of blindness worldwide and the main cause of trichiasis in poor endemic countries^(1,2)^. Trachomatous trichiasis has a natural history distinct from acquired trichiasis, where vision-threatening complications such as corneal opacities and entropion are far more common, as well as reinfection and trichiasis recurrence^(1,2,3)^. However, in developed countries and some emerging countries such as Brazil^(4,5)^, blepharitis now surpasses trachoma as the leading cause of trichiasis, thus increasing the incidence of trichiasis without entropion. Trichiasis can be classified into congenital, trachomatous, or acquired^(6,7)^; isolated or associated with entropion^(4)^ according to the eyelid location (nasal, central, and temporal) or number of segments involved (focal and diffuse)^(4,6)^; or even as major (>5 eyelashes) or minor trichiasis (=5 eyelashes)^(4,5,8)^.

Mechanical epilation is an easy and low-cost procedure for trichiasis; however, owing to its high recurrence rate, it is considered a temporary treatment^(9)^. By contrast, electrolysis, radiofrequency, or cryotherapy can destroy the pathological hair follicle; however, they are locally aggressive, can cause permanent loss of healthy follicles and lid margin scarring, and often recur^(4,9)^. Generally, cryotherapy has a higher complication rate and low success rate^(10,11)^. Argon laser thermal ablation is an in-office procedure that destroys the hair follicle through the skin^(12)^. Nevertheless, it is a costly device, requires lashes with pigment, and can cause lid notching and hypopigmentation^(4,12)^.

Surgery for trichiasis correction still plays an important role in the prevention of visual loss, typically indicated in major or diffuse trichiasis associated with eyelid margin rotation^(2,4,5,13,14)^. Such operation is easy to perform, is low-cost, has a low risk of infection, and has immediate results^(2,4,5,13)^. However, complications such as eyelid deformities and trichiasis recurrence can occur^(15)^. Techniques that fracture the tarsus and rotate the lid margin may injure the meibomian glands and conjunctival goblet cells, further exacerbating symptoms related to dry eye disease^(6,16)^. The interlamellar splitting of the eyelid margin with eversion of the cilia away from the cornea and insertion of a mucous graft were traditionally indicated for moderate-to-severe trichiasis with cicatricial entropion, particularly in the upper eyelids^(17)^. First described by Van Millingen in 1887^(18)^, this technique originally applied the oral transitional cutaneous-mucosal membrane to the superior eyelid. Since then, modifications using different sources of donor grafts have been adapted, and indications have included major trichiasis (>5 pathological eyelashes) without cicatricial entropion, with success rates ranging from 55.0% to 92.6%^(8,17,19,20,21)^. One of its main advantages is the preservation of the eyelid margin anatomy, avoiding damage to the hair follicle roots or tarsus^(17,18)^. Specific to this technique, complications such as trichiasis recurrence, graft failure, and corneal abrasion from the sutures can occur^(17)^.

Currently, fibrin glue is used as a substitute for suturing in a many surgical ophthalmic procedures such as strabismus surgery, conjunctival reconstruction surgery, lamellar corneal grafting for corneal perforation or descemetoceles, and eyelid surgeries^(22)^. To date, no studies have used fibrin glue for trichiasis surgery. Our hypothesis is that the use of fibrin glue for graft adhesion facilitates the interlamellar oral mucosal graft surgical technique and allows application for minor trichiasis cases with failure of conventional treatment. Therefore, the purpose of this study was to describe the outcomes of interlamellar graft surgery with oral mucous membrane graft using fibrin glue in trichiasis at 6-month and 4-year follow-ups.

## METHODS

### Study design and patient population

A prospective study was conducted at the Oculoplastic Division of the Ophthalmology Department of the Ouro Verde Hospital Complex, with the approval of the local ethics in research committee of the Municipal Hospital Dr. Mário Gatti in 2015 (CAAE: 48763015.0.0000.5453). The study adhered to the tenets of the Declaration of Helsinki. The inclusion criteria were patients of age =18 years who presented with trichiasis (major or minor), had failure of =2 previous treatments for trichiasis (i.e. electrolysis, photocoagulation, cryotherapy, and eyelid surgery), were willing and able to undergo surgery, and signed the consent form. The exclusion criteria were patients with associated eyelid margin rotation (i.e., entropion) with or without conjunctival scarring, acute blepharitis or other eye infections at screening visit, and inability or unwillingness to participate in the study.

The patients were evaluated preoperatively to determine the cause, location, and classification of trichiasis and the associated eyelid margin rotation. The cause was classified as acquired, trachomatous, or congenital. In the cases associated with chronic blepharitis, the diagnosis was mainly presumptive, based on patient history and clinical signs such as marginal inflammation or thinning, surrounding crusts on the cilia, and evident meibomian gland dysfunction. If no other associated causes were confirmed, the trichiasis was classified as undetermined. Its location was classified as temporal (lateral one-third of the eyelid), central (middle one-third of the eyelid), and nasal (medial one-third of the eyelid). The trichiasis classification was determined as major (>5 pathological eyelashes) and minor (=5 pathological eyelashes). We advised patients to refrain from manual epilation 2 weeks before surgery to allow identification of the pathological eyelashes and their hair follicle roots during surgery and to determine graft size. The patients were instructed not to rub or touch their eyes and to use a combination of tobramycin and prednisolone ointment twice daily around the operated region during the first week after operation.

For this study, the patients were followed up on day 1, day 7, 1 month, 6 months, and 4 years after operation. On day 1, the patients were evaluated for graft adherence, which was classified as good, when the graft was well adhered to the entire recipient bed; partial, when part of the graft was not adhered or elevated, with focal areas of non-vascularization; or failed, when the graft was no longer on the recipient bed. On day 7, graft adherence and symptom resolution were evaluated; the symptoms included tearing or foreign body sensation, ocular irritation, or pain. On 1- and 6-month after operation, graft adherence, symptom resolution, surgery esthetics, overall patient satisfaction with surgery, and trichiasis recurrence were assessed. The patients classified the esthetics of their surgery as “poor”, “average”, or “good”. They classified their overall satisfaction as “good” when their expectations were met, “partial” when their expectations were partially met, and “unsatisfied” when their expectations were not met. Lastly, the patients were reevaluated 4 years after surgery to assess the graft adherence, symptom resolution, esthetics, overall patient satisfaction with surgery, and trichiasis recurrence.

### Surgical technique

**Figure 1.**
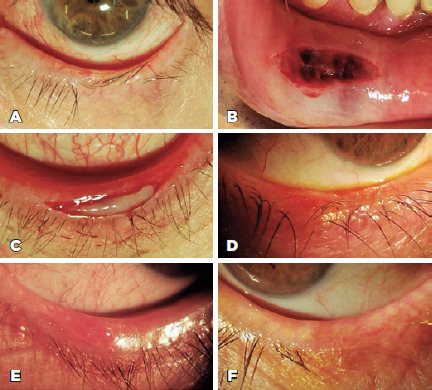
(A) Preoperative photograph of a major trichiasis on the central inferior eyelid. (B) Intraoperative oral mucosal graft donor site. (C) Intraoperative oral mucosa graft placement on the recipient bed. The postoperative photographs of the eye at 1 month (D) and 6 months (E) show good graft adherence and no abnormal eyelashes touching the eye. (F) At 4 years after operation, the patient presented with good graft adherence and focal eyelash discoloration and loss, but no trichiasis recurrence.

After infiltration of the eyelid and conjunctival fornix with 1% lidocaine with 1:50,000 epinephrine, a Desmarres chalazion clamp was used to stabilize the eyelid. An interlamellar incision through the gray line was performed under the microscope, creating a groove (approximately 2 mm deep), which extended 2 mm laterally and medially beyond the area of trichiasis, making sure that all the eyelashes were in the anterior lamella (Figure 1A). Likewise, after local infiltration of the anesthetic agent, an ellipsoidal graft from the inner lower lip mucosa was harvested (Figure 1B) and fitted for insertion in the recipient bed of the eyelid, thereby everting the misdirected lashes away from the eye (Figure 1C). In place of sutures, the fibrinogen solution of the glue was applied underneath the graft, and the thrombin solution, on the receptor bed. The graft was then quickly placed on the recipient bed, allowing a full minute to pass to ensure complete adhesion, and was evaluated at the end of surgery (Supplemental Video). In the advent that the graft was not stable or adhered, sutures would be performed to guarantee graft adhesion. The biological fibrin glue Beriplast P (Aventis Behring, Germany) was used, following all the instructions of the manufacturer. The surgeries were performed by a single surgeon (SVB) who has experience in the technique with sutures.

### Statistical analysis

Descriptive statistics were reported as “mean ± standard deviation” for continuous variables and as “number (percentage from total)” for non-continuous variables. The Kaplan-Meier survival test was performed to determine the cumulative success rate. Chi-square tests were used to determine postoperative symptoms, previous treatment failure, the location and number of eyelid segments involved (temporal, central and nasal), trichiasis classification (minor and major), graft adherence, surgical esthetics, and overall patient satisfaction as risk factors for early (6 months), late (4 years), and overall (total cases) trichiasis recurrence.

## RESULTS

A total of 19 eyes from 15 patients were included, of whom 10 patients (66.7%) were female and 5 (33.3%) were male. The mean age was 75.4 ± 10.5 years (range, 54-98 years). Acquired trichiasis was the main cause in 14 patients (93.3%), of whom 12 (86.7%) showed chronic blepharitis, 2 presented with an undetermined cause (13.3%), and one had trachomatous trichiasis (6.7%). Of the acquired trichiasis cases, 4 eyes (28.5%) showed thinning of the eyelid margin without entropion. A total of 17 eyes (89.4%) had a previous failure of electrolysis treatment, 3 eyes (15.7%) had a previous photocoagulation, and 2 eyes (10.5%) had a previous full-thickness eyelid wedge excision. A total of 3 eyes (15.7%) had failure of >1 treatment modality. The 2 eyes of 2 patients (10.5%) who underwent a previous eyelid surgery showed misdirected eyelashes without entropion at the screening visit. In addition, 10 patients (66.6%) had a history of mechanical epilation 3 months prior to the screening visit. Further, in 2 eyes of 2 patients, a bandage contact lens was required before surgery because of excessive irritation and foreign body sensation from the trichiasis.

The inferior eyelid was the most common location of surgery with graft insertion (89.4%). In 2 eyes of 2 patients (10.5%), the same procedure was performed on the inferior and superior eyelids, where two grafts were harvested, as shown in table 1. Most cases involved only 1 eyelid segment (89.4%), of which the central segment was the most common (63.1%). In addition, most cases presented with minor trichiasis (84.2%). No adverse events occurred during surgery and all grafts were adhered at the end of the surgery; hence, no sutures were required after graft placement. In addition, no adverse reaction to the glue was reported.

**Table 1.** Patient demographics at 6-month follow-up

Patient	Age	Sex	Eye	Eyelid	Segment	Classification	Treatment Failure	Graft Adherence	Symptoms	Surgery Esthetics	Overall Satisfaction	Recurrence
1	82	M	Right	S + I	C	Minor	E	Good	Persisted	Average	PartialAverage	Yes
-	-	-	Left	I	C+T	Major	E, P	Good	Persisted	Poor	Unsatisfied	Yes
2	63	F	Right	I	C+T	Major	E	Good	Resolved	Good	Good	No
-	-	-	Left	I	C	Minor	E	Good	Resolved	Good	Good	No
3	66	F	Right	I	C	Minor	E	Good	Resolved	Good	Good	No
4	74	M	Left	I	C	Minor	E	Good	Resolved	Good	Good	No
5	68	M	Left	I	C	Minor	E	Good	Resolved	Good	Good	No
6	54	F	Right	S + I	C	Minor	E	Good	Resolved	Good	Good	No
7	82	M	Right	I	T	Minor	P	Good	Resolved	Good	Good	No
8	98	F	Right	I	C	Minor	E	Good	Resolved	Good	Good	No
9	74	F	Left	I	T	Minor	E	Good	Resolved	Good	Good	No
10	78	F	Right	I	C	Minor	E	Partial	Resolved	Average	PartialAverage	No
11	83	F	Right	I	C	Minor	E, S	Good	Persisted	Good	PartialAverage	No
-	-	-	Left	I	C	Minor	E, S	Good	Persisted	Good	Good	No
12	68	F	Left	I	T	Minor	P	Good	Resolved	Good	Good	No
13	80	F	Right	I	T	Minor	E	Good	Resolved	Good	Good	No
14	80	M	Right	I	C	Minor	E	Good	Resolved	Good	Good	No
-	-	-	Left	I	T	Minor	E	Good	Resolved	Good	Good	No
15	82	F	Right	I	C	Major	E	Partial	Resolved	Good	Good	No

M= male; F= female; S= superior; I= inferior; C= central; T= temporal; E= electrolysis; S= surgery; and P= photocoagulation.

On the first postoperative day, all the cases showed graft adherence, whereas on postoperative day 7, 2 eyes of 2 patients (10.5%) showed partial graft adherence, showing a focal area of non-vascularization that persisted throughout the entire study visits without progression to graft failure. On day 7 after operation, all the eyes with good graft adherence showed a thin layer of granulation tissue over the graft, which was reabsorbed at the subsequent visits. Moreover, on day 7, 4 eyes of 2 patients (21.0%) showed persistent symptoms, mainly irritation and dry eye sensation, despite the surgical success without any visible misdirected eyelashes. The same patients reported symptoms consistently throughout study visits and no other symptom-free patient developed symptoms at the subsequent follow-up. At 1-month follow-up, the same 17 eyes of 13 patients (89.4%) showed good graft adherence, as shown in Figure 1D. No new symptom, no graft failure, or trichiasis recurrence was reported.

A total of 19 eyes of 15 patients were evaluated at 6-month follow-up, and the patients’ demographics are shown in table 1. Seventeen eyes of 13 patients (89.4%) showed good graft adherence, as shown in Figure 1E. Two eyes of 2 patients (10.5%) showed partial graft adherence and no graft failure. A total of 15 eyes of 13 patients (78.9%) reported symptom resolution, 16 eyes of 13 patients (84.2%) showed good esthetics, and 13 patients (15 eyes, 78.9%) had high overall satisfaction with surgery. At 6-month follow-up, 2 eyes of 1 patient (10.5%) showed trichiasis recurrence, so a full-thickness pentagonal lid margin resection was indicated.

At 4-year follow-up, 3 eyes of 3 patients (15.7%) were lost to follow-up. Therefore, 14 eyes of 11 patients were evaluated, as shown in table 2. No graft failures were observed, and 12 eyes of 10 patients (85.7%) remained with symptom resolution. All 14 eyes of 11 patients (100.0%) reported good esthetics, and 13 eyes of 11 patients (92.9%) reported good overall satisfaction from surgery, with 1 eye of 1 patient showing a change from partial overall satisfaction at 6-month follow-up to good overall satisfaction at 4-year follow-up. Finally, 12 eyes of 11 patients (85.7%) showed good graft adherence, as shown in figure 1F. Two eyes of 2 patients (14.2%) showed trichiasis recurrence.

**Table 2.** Patient demographics at 4-year follow-up

Patient	Age	Sex	Eye	Eyelid	Segment	Classification	Treatment Failure	Graft Adherence	Symptoms	Surgery Esthetics	Overall Satisfaction	Recurrence
1	82	M	Right	S + I	C	Minor	E	-	-	-	-	-
-	-	-	Left	I	C+T	Major	E, P	-	-	-	-	-
2	63	F	Right	I	C+T	Major	E	Good	Resolved	Good	Good	No
-	-	-	Left	I	C	Minor	E	Good	Resolved	Good	Good	No
3	66	F	Right	I	C	Minor	E	-	-	-	-	-
4	74	M	Left	I	C	Minor	E	-	-	-	-	-
5	68	M	Left	I	C	Minor	E	-	-	-	-	-
6	54	F	Right	S + I	C	Minor	E	Good	Resolved	Good	Good	No
7	82	M	Right	I	T	Minor	P	Good	Resolved	Good	Good	No
8	98	F	Right	I	C	Minor	E	Good	Resolved	Good	Good	No
9	74	F	Left	I	T	Minor	E	Good	Resolved	Good	Good	No
10	78	F	Right	I	C	Minor	E	Partial	Resolved	Good	Good	No
11	83	F	Right	I	C	Minor	E, S	Good	Persisted	Good	PartialAverage	Yes
-	-	-	Left	I	C	Minor	E, S	Good	Persisted	Good	Good	No
12	68	F	Left	I	T	Minor	P	Good	Resolved	Good	Good	No
13	80	F	Right	I	T	Minor	E	Good	Resolved	Good	Good	No
14	80	M	Right	I	C	Minor	E	Good	Resolved	Good	Good	No
-	-	-	Left	I	T	Minor	E	Good	Resolved	Good	Good	Yes
15	82	F	Right	I	C	Major	E	Partial	Resolved	Good	Good	No

M= male; F= female; S= superior; I= inferior; C= central; T= temporal; E= electrolysis; S= surgery; and P= photocoagulation.

**Figure 2.**
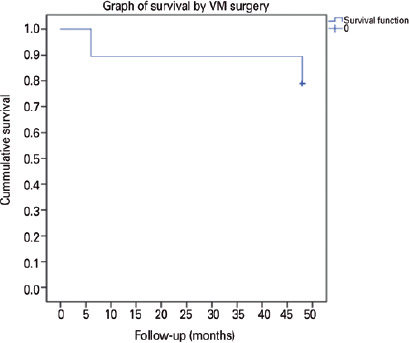
Kaplan-Meier graph of the mucous graft survival after interlamellar oral mucosa graft surgery with fibrin glue for trichiasis, highlighting the cumulative survival rates at 6 and 48 months.

The cumulative success rate after 4 years was 78.9%, with recurrence of trichiasis in 4 eyes of 3 patients. The survival graph of the modified interlamellar oral mucosal graft surgery technique is shown in figure 2. As the symptoms at 1 week after surgery did not change throughout the study, only symptoms at 1 week were considered risk factors of trichiasis recurrence. Therefore, persistency of symptoms after surgery was a significant risk factor of recurrence in the short (6 months; p=0.035) and long terms (4 years; p=0.016). Multiple treatment modality was not a significant factor of trichiasis recurrence (p=0.097). The number of segments and location of eyelid involvement were not significant factors for recurrence (p=0.386 and p=0.563, respectively). In this study, the trichiasis classification was not a significant factor of recurrence (p=0.530). In the eyes with trichiasis recurrence, graft adherence was not a significant factor for recurrence at 6 months or 4 years (p=0.614 and p=0.725, respectively). The reported patient esthetics at 6 months was not a significant factor of recurrence (p=0.065). All the patients reported good esthetics at 4-year follow-up. Furthermore, low overall patient satisfaction at 6-month follow-up was a significant factor of trichiasis recurrence (p=0.009), but not at 4-year follow-up (p=0.143).

## DISCUSSION

Fibrin glue has been used extensively in ocular reconstructive and plastic surgeries^(22,23)^ but not previously reported in trichiasis surgery with interlamellar oral mucosal graft technique. In addition, the long-term results of this surgical technique for trichiasis are scarce in the literature^(24)^. Therefore, we show for the first time, a 4-year outcome of interlamellar splitting and buccal mucosa graft insertion with fibrin glue. Our success rate was 78.9%, which is in accordance to previous results of the technique with sutures^(4,5,8)^.

The underlying cause of trichiasis and its association with eyelid margin misdirection or conjunctival scarring are known to influence the treatment outcome and usually require additional tarsal rotation techniques. Figueiredo et al. highlighted that in cases associated with entropion, the trichiasis recurrence rate increased to 50%; however, in isolated trichiasis, the interlamellar oral graft surgery showed a higher success rate in their case series (92.6%)^(20)^. By contrast, Hirai et al. reported a lower success rate for trichiasis without entropion (58.0%). However, their criterion for surgery was major trichiasis, and their study included severe cases with systemic complications such as Stevens Johnson syndrome, which might have affected the reported outcomes^(8)^. In our cohort, surgery was performed mostly for focal, isolated, and minor trichiasis, with good success rates and no graft failure. Therefore, while surgical treatment is usually indicated for diffuse or major trichiasis^(4,5,8,17)^, our data suggests that this modified interlamellar oral mucosal graft surgery technique should be considered as a second-line treatment for recalcitrant minor trichiasis without concomitant entropion, with good long-term outcomes. This technique has the advantage of preserving the eyelid margin anatomy and decreasing inadvertent damage to the hair follicles or eyelid scarring and deformities, as compared to other treatment modalities^(17)^. For cases with associated entropion, surgery targeting eyelid margin alignment should be indicated.

The proposed surgical technique showed excellent rates of symptom resolution, graft adherence, and esthetic and overall patient satisfaction. Persistency of symptoms after surgery was a significant risk factor of trichiasis recurrence despite good graft adherence. These findings highlight the challenge of treating recalcitrant cases of trichiasis, where recurrence can occur regardless of the technique. In our study, both symptomatic patients had failure in more than one treatment modality. Conversely, the number of previous treatments, classification of trichiasis, number of involved eyelid segments, and location of the trichiasis were not significant factors of trichiasis recurrence in this study. Nevertheless, the overall success rate shows that interlamellar oral mucosal graft surgery with fibrin glue is a viable treatment for major and minor trichiasis. Further, we believe that the dispensation of sutures facilitates the surgical technique, thus allowing for the use of a smaller graft while decreasing the time of surgery and eliminating the discomfort of removing sutures.

In developed and eliminating developing countries, inflammatory diseases involving the eyelids have surpassed the incidence of infectious causes of trichiasis^(4,5)^. Accordingly, our study shows chronic blepharitis as the main cause of acquired trichiasis. In trichiasis associated with chronic blepharitis, central lower eyelid thinning with trichiasis syndrome has been frequently reported^(25)^. In our cohort, 4 eyes (28.5%) with chronic blepharitis presented marginal eyelid thinning. However, no associated entropion was found, as classically described in these patients^(25)^. Hence, our study suggests that the proposed technique could be advantageous for cases of eyelid marginal thinning with trichiasis not associated with entropion.

A recent randomized clinical trial showed that tarsus-sparing surgery was more effective than posterior lamellar tarsal rotation for correcting trachomatous trichiasis of the inferior eyelid^(26)^. We hypothesized that the preservation of the tarsus (without fracture) and anterior lamella in this surgical technique is one of the reasons, if not the main reason, for the success rate. Interlamellar surgery still confers a risk of eyelash loss due to inadvertent damage to the follicle root during the procedure. Therefore, cautious lamellar splitting and graft insertion under the microscope are advised to minimize such risk. Further studies are necessary to determine the effectiveness of interlamellar oral mucosal graft surgery with fibrin glue in non-trachomatous trichiasis by comparing the graft size, different surgical techniques, and treatment modalities.

Furthermore, some authors adopted the cutaneous--mucosal junction graft of the lip to simulate the transition of the palpebral margin with a more rapid integration of the graft and better esthetics than when using the tarsal-conjunctival graft of the upper eyelid^(20)^. The lower lip mucosal graft was chosen because it is simple to execute, leaves no visible scarring, requires no sutures in the donor site, and causes less postoperative discomfort by avoiding the sensitive cutaneous-mucosal transition region. In our opinion, this provides better esthetics because the exposed graft on the outer lid margin undergoes keratinization, thus blending with the neighboring tissue.

The main limitation of this study is the small number of patients treated and the loss to follow-up at 4 years, making it difficult to determine the factors that might have contributed to the recurrence rate with the adapted surgical technique. Nevertheless, to the best of our knowledge, we report the longest follow-up period after interlamellar oral mucosal graft surgery for trichiasis, and the patient dropout rate was within the acceptable range (15.7%). Further studies with larger sample sizes are warranted. In addition, owing to the small number of cases with major trichiasis, whether the modified technique would show different outcomes in major trichiasis remains unclear. However, we believe that the use of fibrin glue should show similar results as those in previous reports, which traditionally indicate interlamellar oral mucosal graft surgery for major trichiasis. From a different perspective, the substitution of sutures with fibrin glue should not interfere with the success rate but rather facilitate the intraoperative technique. Furthermore, the cost of fibrin glue is another limiting factor when compared with the cost of sutures. To overcome this issue, we scheduled several patients on the same day or in conjunction with pterygium surgeries using the biological glue, enabling multiple applications. This is possible, as Osborne et al noted, if the fibrin solution is applied to one surface and the thrombin solution to the another, avoiding the 2-syringe clip application device in the kit, thereby optimizing the glue usage while maintaining precautions to avoid contamination by tissue contact^(23)^. Future studies for comparing the cost-benefit of the surgical modification and other in--office procedures for trichiasis are necessary. Lastly, we did not measure the eyelid thickness before and after interlamellar graft surgery. Although we subjectively noted that the graft tissue undergoes remodeling and shrinkage during wound healing, eyelid thickness changes should be considered in future studies, specifically in cases with eyelid marginal thinning.

In summary, our study shows a good success rate of interlamellar oral mucosa graft surgery using fibrin glue for the treatment of trichiasis after 4 years, with shorter surgical time, good symptom resolution rate, high overall patient satisfaction, and good esthetics. Furthermore, persistent symptoms after surgery were a risk factor of recurrence in this study. In conclusion, this modified surgical technique is a good treatment option for trichiasis without entropion and should also be considered in recalcitrant cases with focal and minor trichiasis.
